# UniCor and UniCorP: a novel metric and hierarchical feature selection algorithm for microbial community analysis

**DOI:** 10.1093/ismeco/ycaf174

**Published:** 2025-09-27

**Authors:** Sebastian Staab, Kim-Isabelle Mayer, Anny Cárdenas, Raquel S Peixoto, Falk Schreiber, Christian R Voolstra

**Affiliations:** Department of Biology, University of Konstanz, Konstanz 78457, Baden-Württemberg, Germany; Department of Biology, University of Konstanz, Konstanz 78457, Baden-Württemberg, Germany; Department of Biology, University of Konstanz, Konstanz 78457, Baden-Württemberg, Germany; Department of Biology, American University, Washington DC 20016, United States; Red Sea Research Center (RSRC), Biological and Environmental Sciences and Engineering Division (BESE), King Abdullah University of Science and Technology (KAUST), Thuwal 23955, Makkah Province, Saudi Arabia; Department of Computer and Information Science, University of Konstanz, Konstanz 78457, Baden-Württemberg, Germany; Faculty of Information Technology, Monash University, Clayton Victoria 3800, Australia; Department of Biology, University of Konstanz, Konstanz 78457, Baden-Württemberg, Germany

**Keywords:** hierarchy, feature selection, feature aggregation, machine learning, artificial intelligence, propagation, microbiome, taxonomy, correlation, metric

## Abstract

The rapid advancement of technologies and methods in the life sciences has significantly increased the availability of big data, presenting new challenges for its analysis. Microbiome datasets, in particular, are characterized by extensive feature sets with defined but complex hierarchical structures that are often overlooked or underutilized. Here we introduce a novel metric, UniCor, to identify UNIquely CORrelated eNtities (UNICORNs) in quantitative datasets associated with continuous target variables. These datasets may include microbiome community structures in relation to environmental factors (e.g., temperature, pH, salinity) or biotic variables (e.g., thermal tolerance, oxidative stress). The UniCor metric combines the uniqueness of a given feature within a dataset with its correlation to a target variable of interest. To further enhance its utility, we developed a propagation algorithm (UniCorP), which exploits inherent dataset hierarchies, such as taxonomic levels in microbiome datasets, by selecting and propagating features based on their UniCor metric. Using bacterial community datasets with hierarchical taxonomic annotations and various continuous environmental variables, we demonstrate the ability of the novel metric to reduce features and increase predictive performance in cross-validated Random Forest Regressions. After propagating features with UniCorP and enriching the hierarchical levels with UNICORNs, the predictive performance consistently outperformed control trials for taxonomic aggregation, even at the least granular hierarchical level, allowing a substantial reduction of the feature space. We also compared the metric to existing methods for feature aggregation, showing that it offers stable, competitive predictive performance and feature reduction, within a simple and adaptable framework.

## Introduction

With technological advancements in the life sciences, new and larger datasets are being generated, particularly in high-throughput omics fields like microbiome research, presenting both challenges and opportunities for information mining and computational analysis. Hierarchies within datasets present one of the opportunities that often remain unused in classical machine learning (ML) approaches or analytical pipelines. These hierarchies can be inherent to different parts of a dataset (e.g., in the feature set or accompanying measured variable) and can appear in different computations (e.g., in classification or regression tasks). The application of ML methods to facilitate hierarchical classification in biological datasets was discussed by Rezende *et al.* [1]. Similarly, Wan and Freitas [2] compared hierarchical feature selection methods in classification tasks applied to Gene Ontology (GO) datasets using binary features, while Frisby *et al.* [3] developed Harvestman, a hierarchical feature learning and selection tool with a similar goal.

A prominent type of biological data that typically features inherent hierarchies is microbiome data, i.e., community matrices [4]. In this context, hierarchy refers to the tree-like taxonomic structure that organizes microbial features across multiple levels, with each taxon nested within a single parent group—such as species within genera, genera within families, and so on. Microbiomes connect all living entities on our planet and play a fundamental role in shaping ecosystems as we know them [5, 6]. Microbiomes influence human and environmental health, making them a key target for developing solutions to pressing global challenges such as disease, climate change, and ecosystem sustainability [7]. Thus, understanding the key features associated with their beneficial roles is paramount. Recent efforts have focused on exploring ML methods for microbiome research. For instance, the European Cooperation in Science and Technology Action “ML4Microbiome” initiative was launched to tackle existing limitations in this area [8]. Reviews in the field have highlighted key developments, including an updated overview of ML approaches tailored to microbiome research [9] and statistical methods, including abundance analysis, integrative analysis, and network analysis [10]. More recent efforts have explored the use of ML in longitudinal (time-series) analysis of microbiome data, providing a comprehensive view of the evolving landscape of tools and techniques [11]. Despite these advancements, inherent hierarchies within microbiome data—such as those derived from taxonomic annotation of prokaryotic entities—remain largely overlooked.

Microbiome sequencing datasets implicitly represent a hierarchical structure through the taxonomic classification of microbial taxa. For example, 16S rRNA (or other) marker gene microbiome datasets create high-dimensional feature spaces where each feature corresponds to a distinct microbial taxon, often represented as Amplicon Sequence Variants (ASVs) or Operational Taxonomic Units (OTUs) [12]. These datasets are typically sparse and wide, containing a large number of either small values, zeros, or missing entries due to the long-tailed distribution commonly observed in microbial communities, where few bacterial taxa are highly abundant while many are rare [13]. Leveraging the underlying taxonomic hierarchy has the potential to reduce feature space dimensionality, minimizing noise and redundancy, while producing more refined (focused) and interpretable datasets.

Although prominent methods such as LEfSe [14] and ANCOM [15] focus on differential abundance testing across categorical groups, they do not leverage taxonomic structure. However, several more recent approaches have begun to incorporate hierarchical information into their statistical frameworks. For instance, Phylofactorization [16] iteratively partitions a phylogenetic tree to identify patterns across phylogenetic scales, while treeClimbR [17] analyzes hierarchical trees at different resolutions and pinpoints branches or leaves containing features of interest. Other methods, such as StructFDR [18] and miLineage [19], use tree-based smoothing or lineage-level modeling to improve power and control for false discoveries. Although these tools exploit biological hierarchies, their primary aim is statistical inference rather than feature selection or dimensionality reduction. In contrast, SHSEL [20] is a method that eliminates redundant features along inherent hierarchical paths of the feature set, followed by pruning the remaining features based on their relevance to a classification task. To measure the similarity in relevance between two binary feature nodes, the authors employed the information gain metric [21] or Pearson’s correlation coefficient. Another approach tailored to microbiome research is the hierarchical feature engineering (HFE) algorithm [22] that improves sample classification accuracy by reducing the feature space. Inspired by SHSEL [20], the HFE approach first represents higher taxonomic units by summing the relative abundances of their child nodes. Then, features with a Pearson’s correlation coefficient above a threshold between child and parent nodes are propagated to the next higher level. The hierarchical tree is then pruned based on the information gain metric accounting for features with incomplete information. SHSEL and HFE are designed for classification tasks and can handle categorical observed variables [20, 22]. This concept can be expanded by incorporating methods for continuous observed variables [23]. The TaxaHFE algorithm begins with tree construction, followed by a feature filter based on abundance and prevalence, before collapsing child-features based on their correlation to their parent-feature. Next, the feature importance of child- and parent-features is measured by using a Random Forest model, guiding the feature set reduction. An optional final Random Forest model considers all selected features and drops those with low importance. Applied to microbiome datasets, TaxaHFE reduced the feature count by 90% on average while maintaining predictive performance, showcasing its effectiveness in enhancing model performance and interpretability [24]. A related method, TASSO [23], formulates hierarchical feature selection as a regularized regression problem. It applies a tree-guided fused lasso penalty within a log-contrast regression framework to identify subcompositions that are predictive of a continuous target variable. The fused lasso encourages similar coefficients for closely related taxa in the hierarchy, effectively shrinking uninformative features and merging correlated ones. This yields a sparse, hierarchy-aware selection of features. However, in contrast to TaxaHFE’s heuristic approach, TASSO depends on model-specific regularization and parameter tuning, which may limit its broad usability across different modeling frameworks.

As outlined, although methods for hierarchical feature selection in microbiome research exist, the field remains relatively new and open. Most existing methods are either not tailored to microbiomes [20], are limited to categorical target variables [22], or ignore taxonomic structure [14, 15]. Others incorporate taxonomic hierarchies, but focus primarily on statistical testing across taxonomic levels [16–19] rather than enriching and aggregating feature sets for predictive modeling. More recently, methods such as TaxaHFE [24] and TASSO [23] have addressed continuous target variables, leveraging taxonomic hierarchies through multi-step filtering and tree-guided regularization, respectively. However, these methods either depend on embedded ML models or require model-specific tuning procedures. Ideally, a simple, robust, and fast approach is desired—one that enables hierarchy-aware feature selection while remaining model-agnostic and easy to integrate into diverse data analysis pipelines.

To provide this, we developed UniCor and UniCorP. UniCor is a new metric that combines the correlation to a continuous target variable of interest with the uniqueness of a feature within the feature set, i.e., a microbial taxon in a microbiome dataset. We define uniqueness as the property of being strongly correlated with the target variable while being minimally correlated to other features in the dataset, making them nonredundant predictors. To complement the UniCor metric, we devised the UniCorP algorithm that exploits feature set hierarchies (i.e., taxonomic annotation of a microbial entity) by employing the UniCor metric to identify and propagate UNIquely CORrelated eNtities (UNICORNs). We demonstrate the advantages of UniCorP over baseline aggregation across the different taxonomic levels and compare it to TaxaHFE, an algorithm that reduces taxonomically annotated microbiome datasets based on correlation and feature importance [24]. UniCor requires a quantitative feature set with an unambiguous hierarchy connected to a target variable of interest. Although continuous target variables are typically used for regression tasks, any quantitative target variable may be used. A careful selection of target variables is thus recommended, as discrete (discontinuous) target variables violate the assumptions of linearity for regression tasks. To assess the performance of UniCor, we employ microbial community matrices from coral samples in conjunction with host physiological data, e.g., standardized thermal tolerance threshold (ED50, effective dose 50, i.e., the temperature at which initial performance is reduced by 50%) or SST (sea surface temperature) target variables, respectively [25–27].

## Materials and methods

### UniCor—metric

To compare features within a hierarchy to all other features and the target variable, both of which are quantitative, a new metric is needed. We propose the UniCor metric to identify so-called UNICORNs, which represent features that are unique and putatively important predictors of the target variable.

To compute such a score, a correlation method must first be selected. UniCor supports both Pearson and Spearman correlations. Pearson measures linear relationships and assumes continuous, normally distributed variables, while Spearman is rank-based and more robust to nonlinear or non-normally distributed data.

To account for the compositional nature of microbiome data and potential biases in feature magnitude, UniCor offers optional data transformations prior to metric computation. Specifically, the input abundance matrix can be used as-is, normalized to relative abundances, or transformed using the centered log-ratio (CLR) approach [28]. The CLR transformation is a popular method in compositional data analysis that projects the data into real space while preserving ratios between features, thereby facilitating meaningful correlation analyses. While relative abundance and raw count inputs result in identical rank orderings and correlation metrics, CLR-transformed data may reveal different patterns by adjusting for total-sum constraints.

The UniCor metric is computed by the absolute correlation between the respective feature and the target variable subtracted by the average feature–feature correlation of said feature, as presented in equation (1):


(1) \begin{equation*} UniCor\ Metric\ of\ featur{e}_i=\frac{\vert fc{c}_i\vert -\overline{ff{c}_i}}{2}, \end{equation*}

where $\vert fc{c}_i\vert$ is the absolute feature–target variable correlation and $\overline{ff{c}_i}$, the average feature–feature correlation between $featur{e}_i$ and all other features. Dividing by 2 normalizes the resulting scores: Due to $\vert fc{c}_i\vert$ taking on values between 0 and 1 and $\overline{ff{c}_i}$ taking on values between −1 and 1, this limits the UniCor metric to values between $-\frac{1}{2}$ and $1$. Features with a higher absolute (positive or negative) feature–target correlation score higher than features with a correlation closer to zero. Features with a positive average feature–feature correlation are less unique and score lower than features with a negative feature–feature correlation. The computational complexity of $\overline{ff{c}_i}$ approximately follows $O(nm2)$ for creation of a whole correlation matrix. Here *n* is the number of samples and *m* is the number of features. For $\vert fc{c}_i\vert$ the correlation has to be computed only once for each feature, following $O(nm)$. If the Spearman correlation is used, an additional $O\left( mn\ \mathit{\log}\ n\right)$ sorting step is required and implemented to rank-transform the data, but in cases where $m>>n$, this does not affect the leading complexity term. Thus, $\overline{ff{c}_i}$ is dominating the computational complexity leading to a computational complexity of $O(nm2)$ for the UniCor metric. Our approach represents a simple metric, i.e., easily computable, intuitively interpretable, and comparable between features of the same feature set. Of note, for large feature sets the average feature–feature correlation will converge to the same value for all features. Thus, the possibility to group or cluster features in large and sparse feature spaces can help to retain the information value of the UniCor metric. The hierarchical structure of microbiome datasets represents such inherent clusters (taxonomic groups) and can be utilized. In the following section we present an algorithm, UniCorP, that can be used to run through multiple levels successively, utilizing UNICORNs progressively, while traversing through the taxonomic hierarchy in a bottom-up approach.

### UniCorP—bottom-up UniCor propagation

Typical microbiome datasets exhibit a sparse and wide nature, consisting of a relatively small number of samples but thousands of features. This is due to the long tail distribution commonly observed in microbial communities, where many bacterial taxa have zero or near-zero abundances (i.e., are rare), while a few are highly abundant. This “curse of dimensionality” makes it difficult to detect meaningful patterns and extract relevant features due to noise and redundancy. The taxonomic hierarchy inherent in microbiome datasets allows for aggregating features into groups, thus reducing the feature space dimensionality. Our proposed novel approach is based on the circumstance that the hierarchical grouping of microbiota is not "perfect", as not all bacterial species within the same taxonomic group exhibit similar functions or are perfectly correlated. Some features might represent keystone species or be specifically relevant to the target variable. Simply aggregating according to the hierarchy poses the risk of losing or diffusing this distinctive information. The UniCor metric enables the detection of such potentially important features, while the Bottom-Up UniCor Propagation Algorithm (UniCorP) propagates them to higher hierarchical levels. This allows for an efficient and meaningful reduction of the feature space while retaining relevant information.

Three data files are needed as input for the UniCor metric and UniCorP algorithm (Table 1): First, the continuous target variable of *n* samples should be provided as a single column with unique sample IDs as row indexes (Supplementary File S1). Second, a quantitative feature set (i.e., microbiome community data matrix) should be prepared with *n* matching sample IDs as row indexes and unique feature IDs as column headers for *m* features (Supplementary File S2). If binary classes represent (some of) the features, they can be transformed to [0,1] in order to be used like quantitative features. Third, the hierarchy file (i.e., taxonomic annotation of features), with *m* matching feature IDs as column headers and the respective hierarchical annotations as text values, i.e. strings (Supplementary File S3, Table 1).

**Table 1:** Dimensions of input files. *n* = number of samples, *m* = number of features, *l* = number of hierarchical levels.

input file	dimensions: rows^*^columns
continuous target variable	n^*^1
quantitative feature set	n^*^m
hierarchical annotation	l^*^m

Of note, the hierarchical annotation must be tree-structured, meaning that each child node has only one parent node, and the number of nodes decreases toward higher levels. Hierarchies with a flat structure (e.g., one child per parent across levels) provide no exploitable information. While taxonomic annotations typically fulfill this requirement, certain other omics hierarchies such as KEGG or GO may form directed acyclic graphs, where features can have multiple parents or remain disconnected. These are not supported by UniCorP.

To determine which features (i.e., UNICORNs) are propagated to the next higher (less specific) hierarchical level, UniCorP offers two mutually exclusive selection strategies. One option applies a threshold to the UniCor score, retaining only features that exceed this value at each given hierarchical level. The threshold for feature propagation to the next level can be set by the user. We recommend using threshold values between 0 and 1. Values closer to 0 propagate more features to the higher hierarchies, while values closer to 1 are more restrictive. Negative threshold values (up to −0.5) are possible but are rarely insightful, as large datasets with multiple features generally contain some positive UniCor metric scores. The optimal choice depends on the dataset’s inherent correlations and the user’s required or wanted feature set size, which might necessitate empirical testing using various thresholds. In general, less restrictive thresholds increase the number of features at the cost of lesser correlation. Alternatively, users can specify a *top_k* parameter to keep the *k* highest-scoring features per level. While thresholding allows dataset-specific sensitivity and interpretability, the *top_k* setting offers more control over the resulting feature dimensionality and thus, computational complexity.

**Figure 1:**
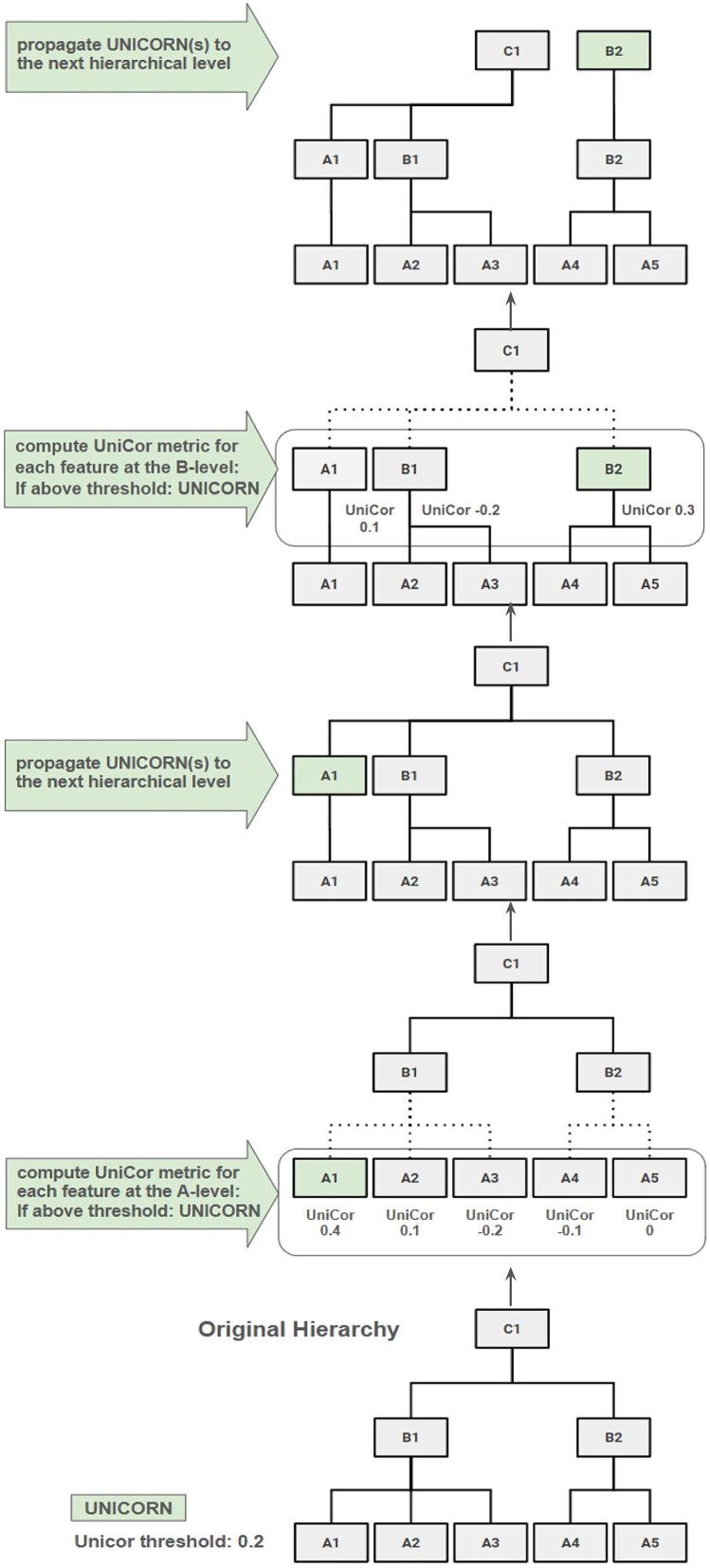
Bottom-up UniCor propagation (UniCorP). The tree visualizes a section of a hierarchy (e.g., a phylogenetic tree) where C1 is the parent node to B1 and B2. B1 is the parent node to A1 to A3, while B2 is the parent node to A4 and A5. UniCorP works in a bottom-up approach starting from the lowest (i.e., most specific) A level and iteratively propagating UNICORNs (i.e., features that exhibit an above threshold feature–target correlation in comparison to feature–feature correlations) to the next higher/subsequent level, i.e., from A to B, from B to C, etc. until the highest level is reached. Propagated UNICORNs replace their original parent node by becoming their own parent node and thus representing themselves at the next higher level. With an example threshold of 0.2, features that exceed this threshold (i.e., UNICORNs) are being propagated to the next level. Features with UNICOR metrics below the threshold will not be propagated but will be represented by their parent node (A4 and A5 as B2). Out of all features on the lowest level, A1 has a UniCor metric surpassing the threshold and thus gets propagated to the next higher level (B-level, A1 to A1). A2 and A3 are represented as B1. After a new computation of the UniCor metric at the B level, B2 surpasses the threshold and gets propagated to the highest level (C-level, B2 to B2). The previous UNICORN A1 and B1 are represented as C1.

The UniCorP algorithm starts by computing the UniCor metric for all features at the lowest hierarchical level with all features grouped according to their parent node (of the next higher hierarchical level). For each feature *i*, $ff{c}_i$ is computed based on the average correlation to all features in the same group/parent node (e.g., all species within the same genus). If the metric surpasses the specific threshold, the respective feature is propagated to the next higher level. This process repeats until the highest hierarchical level is reached. Since the metric has to be computed repeatedly for every level, UNICORNs can be propagated once or multiple times through subsequent levels if they are repeatedly detected as UNICORNs. This is to say that outstanding features that uniquely correlate to the target variable at each level will be retained and propagated to the highest level (Figure 1).

#### Algorithm pseudocode: UniCorP—bottom-Up UniCor propagation

Let X ∈ ℝ^nxm^ be the feature matrix (samples × features), y ∈ ℝ^n^ the continuous target variable, and H = {H₁, H₂, ..., H_L_} a strict hierarchy from the lowest level H₁ to the highest level H_L_, transformation ∈ {None, relative_abundance, clr} be the choice of transformation, method ∈ {pearson, spearman} be the correlation metric for the UniCor metric computation, and selection method ∈ {threshold θ, top_k} be the method to select UNICORNs.

Algorithm (for each level l from 1 to L − 1):

1. Aggregate features in X by H_1_: X_l_ = aggregate(X, group_by = H_1_)
2. If using transformation: apply *CLR* or *relative_abundance* transformation
3. For each parent group G ∈ H_l + 1_:
  - Identify the child features f_1_, ..., f_j_ ∈ H_l_ that belong to group G
  - Form a group-specific correlation matrix among {f₁, ..., f_j_} and target y
  - Compute UniCor score for each child feature f ∈ G:
    UniCor(f) = 0.5 ^*^ |corr(f, y)| - 0.5 ^*^ mean({corr(f, f’) | f’ ∈ G, f’ ≠ f})
4. Select features (UNICORNs):
  - If using threshold θ: select f if UniCor(f) > θ
  - If using top_k: sort features by UniCor score and select top k features
5. Propagate selected features by assigning their parent group in H_l + 1_ to themselves: H_l + 1_[f] ← f

Output: updated hierarchy H′ enriched with selected informative features at higher levels.

In a worst case scenario, for every level of *m* hierarchical levels (every level the number of nodes is reduced by one) all features are selected as UNICORNs and propagated, leading to $O(nm3)$ with *n* being the number of samples and *m* being the number of features. However, the “grouping” of features can improve the runtime complexity of the algorithm, since the correlation matrix doesn’t have to be computed for the full feature set but rather for the groups independently. Under the assumption of a wide hierarchical tree (e.g., number of hierarchical levels << *m*) and a restrictive UNICORN selection (not every feature gets propagated), we expect $O\left(n{m}_{Gmax}2\right)$ with ${m}_{Gmax}$ being the number of features in the largest group. In big biological datasets with ${m}_{Gmax}<<m$ (e.g., many groups of roughly equal size), this yields a significant reduction in runtime complexity over a single computation $O(nm2)$ of a typical correlation matrix.

The result of the UniCorP algorithm is a newly created hierarchy with the higher levels being enriched by UNICORNs of the lower levels. According to the researchers’ needs, each level of the hierarchy can be accessed and used for further exploration.

## Results and discussion

In a pilot run, we applied the UniCorP algorithm to a microbiome dataset comprising 28 *Stylophora pistillata* coral colonies sampled across four sites in the Red Sea and their corresponding thermal tolerance thresholds (ED50 values) [25]. The dataset includes bacterial abundance data combined with a physiological target variable. The continuous ED50 values indicate the temperature at which a coral colony retained half of its initial algal photosynthetic efficiency, serving as a proxy for thermal tolerance [29–31]. The bacterial abundance data are presented as quantitative count values for the determined ASVs and their associated taxonomic hierarchies, utilized by UniCorP starting from the ASV level (Table 2). Missing annotations in the taxonomic hierarchy are filled with their respective ASV identifier at all taxonomic levels. We systematically evaluated all logical combinations of correlation metrics (Spearman, Pearson) and data transformations (none, relative abundance, CLR) within UniCorP, across a range of propagation parameters (*thresholds* and *top_k* values).

**Table 2:** Comparison of predictive performance (R^2^ and MSE) between common Phylum-level aggregation and UniCorP-based feature aggregation on the *Stylophora pistillata* dataset (28 samples, 4,568 ASVs)**.**

**Method**	**Transformation**	**Threshold**	**Top_k**	**R** ^**2**^	**MSE**
phylum_aggregation				0.532	1.227
Spearman			150	0.621	0.995
Spearman		0.175		0.617	1.006
Pearson			200	0.6182	1.002
Pearson		0.1		0.594	1.066
phylum_aggregation	relative_abundance			0.503	1.304
Spearman	relative_abundance		50	0.608	1.028
Spearman	relative_abundance	0.2		0.631	0.969
Pearson	relative_abundance		100	0.59	1.075
Pearson	relative_abundance	0.1		0.619	1.001
phylum_aggregation	clr			−0.029	2.701
Pearson	clr		10	0.427	1.504
Pearson	clr	0.15		0.328	1.764

Shown are *phylum_aggregation* performance metrics as a baseline for the respective transformation as well as the top performing models for every combination of correlation metric (method), transformation, and either *threshold* or *top_k* as parameter for UNICORN selection (Supplementary File S4). Predictive performance (R^2^ and MSE) was assessed via leave-one-out cross-validation, where in each run, UniCorP-based feature aggregation and transformation were applied to the training data, a Random Forest Regressor was trained on the resulting features, and performance was evaluated on the held-out test fold.

Other correlation metrics such as SparCC [32], Spiec-Easi [33], and HARMONIES [34] focus on inferring internal feature–feature networks under compositional constraints. However, they are unsupervised, not designed to capture feature–target associations, and computationally too complex for UniCor’s lightweight, interpretable scoring.

Random Forest Regression (RFR), widely regarded as the gold standard in microbiome host trait prediction [35], served as a benchmark. We used the RandomForestRegressor implementation from the scikit-learn Python library (v1.3.0) with default parameters, n_estimators = 100 (number of trees) and random_state = 42 (seed). To evaluate model performance, we used two standard regression metrics—R^2^ and mean squared error (MSE)—which quantify the proportion of variance explained and the average prediction error, respectively. These values assess how well microbiome-derived features predict coral thermal tolerance (ED50). For additional, robust microbiome feature analysis using a variety of ML models, we recommend Coracle [4]. Both UniCorP propagation and downstream RFR were jointly leave-one-out cross-validated. We tested two evaluation pipelines: one where the RFR was applied to the transformed datasets (matching the input used in UniCorP) (Supplementary File S4) and another where only UniCorP operated on transformed data while RFR was trained on the original (untransformed) count data (Supplementary File S5)—both enriched with propagated UNICORNs.

Across all combinations, the best performance (R^2^ = 0.631) was achieved using Spearman correlation with relative abundance-transformed data and a threshold of 0.2 (Table 2). However, multiple configurations performed similarly well (R^2^ ~ 0.60), indicating that UniCorP is robust across different correlation methods and parameter choices. Most UniCorP-enriched feature sets clearly outperformed their corresponding phylum-aggregated baselines (Supplementary File S4). Notably, CLR transformations led to markedly lower R^2^ values when used as input for RFR when compared to other method-transformation combinations (Supplementary File S4) or training the same (CLR) enriched datasets on their untransformed (count values) representation (Supplementary File S5). This effect aligns with previous findings that tree-based models, such as Random Forests, are robust to raw count data and may be negatively impacted by log-ratio transformations, which can distort feature relationships or amplify noise in sparse microbiome datasets [36]. Nonetheless, UniCorP substantially improved performance over phylum-level aggregation even when microbial abundance data was CLR-transformed, with the best R^2^ increasing from −0.029 to 0.427. As a result, while CLR-transformed input may not be ideal for tree-based models, UniCorP can still enhance downstream performance in these cases.

For further analyses, we selected *threshold* = 0.15 and *top_k* = 150, as both settings yielded consistently strong predictive performance across combinations of transformation and correlation methods. We recommend the use of the *top_k* parameter for most users, as it offers intuitive control over the resulting feature set size and generally produces more stable results. Based on our results, Spearman correlation emerged as the most robust metric and is therefore recommended as the default choice for microbiome datasets or datasets of similar sparsity and compositionality.

In addition to the pilot run, we chose to test UniCor on the CDIV (coral diversity) dataset from the *Tara* Pacific expedition comprising 2,470 coral samples [27, 37, 38]. The dataset consists of ASV bacterial abundance data of corals collected across the Pacific, with each sample being associated with extensive environmental metadata [26]. The community matrix (i.e., ASV abundance table) was built using DADA2 v1.14 as described in the published scripts [38]. After removal of putative contaminants and eukaryotic ASVs, coral host annotation was provided to the genus level and samples were associated with their respective environmental metadata based on sampling station [39]. UniCor was run on bacterial abundance data generated from four coral genera *Acropora*, *Montipora*, *Pocillopora*, and *Porites* with SST at collection sites serving as the continuous target variable. Samples without corresponding SST values were excluded and bacterial ASVs without abundance in the dataset of the respective coral genus were removed. The four resulting datasets are shown in Table 3. In order to receive an unambiguous hierarchical annotation, missing values within the taxonomy were filled with the next available (hierarchically higher) annotation. In this case, the taxonomy included the ASV level as the lowest and most detailed hierarchical level.

**Table 3:** Number of samples and number of bacterial ASVs (features) for each of the four most common coral hosts (genus level) of the CDIV *Tara* Pacific dataset.

**Coral host**	**No. of samples**	**No. of bacterial ASVs**
*Acropora*	220	48,877
*Montipora*	121	96,520
*Pocillopora*	100	27,792
*Porites*	82	50,824

Following testing of the different method-transformation combinations on *threshold* = 0.15 and *top_k* = 150 in a 10-fold cross validation (Table 4), UniCorP showed similar, albeit even clearer improvements for predictive performance over taxonomic aggregation and smaller, more focused feature sets than without any feature aggregation for all four coral genera (Supplementary File S6).

**Table 4:** Comparison of predictive performance (R^2^ and MSE) between common Phylum-level aggregation and UniCorP-based feature aggregation (relative abundance, Spearman, top_k = 150) on the CDIV datasets.

**Dataset**	**Method**	**Transformation**	**Top_k**	**R** ^**2**^	**MSE**
*Acropora*	phylum aggregation	relative_abundance		0.353	7.098
*Acropora*	UniCorP Spearman	relative_abundance	150	0.595	4.447
*Montipora*	phylum aggregation	relative_abundance		0.125	6.669
*Montipora*	UniCorP Spearman	relative_abundance	150	0.448	4.204
*Pocillopora*	phylum aggregation	relative_abundance		0.029	7.477
*Pocillopora*	UniCorP Spearman	relative_abundance	150	0.516	3.728
*Porites*	phylum aggregation	relative_abundance		−0.16	6.571
*Porites*	UniCorP Spearman	relative_abundance	150	0.236	4.326

Across datasets, UniCorP outperformed the phylum aggregation baseline by ΔR^2^ = 0.36 on average (SD 0.11). This advantage is supported by a one-sided paired t-test (t = 6.92, *P* = 0.0031) and a bootstrap 95% CI for the mean gain (0.28–0.45) that lies entirely above zero. The *top_k* parameter further demonstrated its utility as the more intuitive and consistent selection criterion (Supplementary File S6). These results support our core claims about UniCorP: it enables substantial performance gains, while producing a focused feature set through a lightweight, model-agnostic process, i.e., it is well-suited as a feature selection step for downstream ML analyses. Of note, UniCorP does not assess statistical significance or control for false discoveries. Rare taxa with limited prevalence may receive high UniCor scores due to chance correlations.

Table 5 shows a runtime comparison of the feature aggregation of phylum-level, TaxaHFE-, and UniCorP-based aggregations to the highest taxonomic level (Phylum) for all four coral genera. The “runtime” is assessed as “out-of-the-box” runtime on a commercially available laptop equipped with an AMD Ryzen 55 500 U (6 cores, 2.1 GHz) and 8GB of RAM.

**Table 5:** Comparison of runtime of three approaches for feature aggregation/feature selection.

	Runtime		
Coral host	Phylum	TaxaHFE	UniCorP
*Acropora*	2.4 s	>1 h	5 min
*Montipora*	4.5 s	>1 h	14 min
*Pocillopora*	1.3 s	40 min	2 min
*Porites*	0.6 s	>1 h	6 min

Phylum denotes phylum-level aggregation without any specific feature selection; TaxaHFE denotes aggregation using TaxaHFE [23]; UniCorP denotes aggregation using the here-presented UniCorP algorithm using the UniCor metric.

The runtime reflects the computational complexity of a single application of each of the three approaches: aggregation to the phylum-level completes within seconds, the intermediately complex computation and propagation of UNICORNs averages a few minutes, and the TaxaHFE algorithm takes ~1 h. While these differences are significant, runtimes of around 1 h are still feasible for datasets of this dimension. Unless the sample and feature sizes get significantly bigger or cross-validated testing and parameter optimization for further computational analysis are required, all three approaches are employable. Test results for TaxaHFE show mostly comparable predictive performance (Supplementary File S7); however, they must be interpreted with caution. The method introduces redundancy by retaining multiple hierarchical representations of features and disrupts compositional integrity by removing features without preserving the full taxonomic structure. Additionally, it uses Random Forest models for feature selection and subsequent evaluation, which may lead to model selection bias. Without joint cross-validation, this setup also suffers from data leakage, collectively limiting the reliability of downstream ML analyses.

By comparison, UniCorP provides a similarly focused feature set and achieves higher predictive performance through a simpler, faster, and fully correlation-based approach. Although propagating and selecting features inherently introduce some bias, UniCorP avoids embedded ML models or feature filters, preserving the dataset’s compositional structure and maintaining model agnosticism. Its computational efficiency makes it fast enough for integration into cross-validated pipelines, supporting reliable downstream analyses and ML workflows in microbial or other hierarchical datasets.

## Conclusion

With the increasing capacity of high throughput technologies, biological datasets are becoming increasingly larger, making feature selection a priority. Here, we introduced UniCor as a method to identify features that uniquely correlate to a continuous target variable of interest. The UniCorP algorithm takes advantage of available hierarchies, such as taxonomic annotations in microbial community datasets. We show the value of this algorithm in providing a more focused feature space and simultaneously improving predictive performance using two real-world datasets, linking coral microbiome to thermal tolerance and sea surface temperature. The UniCor metric is easy to compute and intuitive to interpret, offering a novel combination of information, including feature relevance (correlation with the target variable) and feature uniqueness (correlation to other features). The UniCorP algorithm uses these properties to allow for an effective feature aggregation without losing important information. This approach allows sparse, wide datasets to be condensed into more focused feature sets, opening up access to a broader range of downstream ML algorithms and leading to a decreased computational complexity, a faster work process, and putative new insights. The UniCorP algorithm can be used with all hierarchical datasets that fulfill the conditions of being of quantitative nature and having a strict tree-like hierarchy. The best examples are microbial abundance datasets with a strict taxonomic hierarchy. Similar hierarchies occur outside the biological realm, e.g., geographic data (e.g., city < state < country), time series data (e.g., day < week < month < year), or product classification (e.g., product < subcategory < category), where the identification of UNICORNs and a significant reduction in feature space can be highly valuable for data analysis and management.

## Supplementary Material

supplementary_files_ycaf174

## Data Availability

Data derived from a source in the public domain and partially incorporated in online supplementary material.

## Availability and requirements

**Project name:** UniCor

**Project home page:**  https://github.com/SebastianStaab/UniCor

**Web Implementation:**  www.micportal.org (results are emailed to the address provided on the submission form)

**Operating system(s):** Platform independent

**Programming language**: Python

**Other requirements:** Python ≥3.9, pandas ≥2.0.3, numpy ≥1.23.5, scikit-bio ≥ 0.6.3

**Python package:** pip install unicor

**License:** MIT License
